# Does Measurement of First-Order and Heterogeneity Parameters Improve Response Assessment of Bone Metastases in Breast Cancer Compared to SUV_max_ in [^18^F]fluoride and [^18^F]FDG PET?

**DOI:** 10.1007/s11307-018-1262-3

**Published:** 2018-09-24

**Authors:** Gurdip K. Azad, Francois Cousin, Musib Siddique, Benjamin Taylor, Vicky Goh, Gary J. R. Cook

**Affiliations:** 1 Department of Cancer Imaging, School of Biomedical Engineering & Imaging Sciences, King’s College London, Lambeth Wing, St Thomas’ Hospital, Westminster Bridge Road, London, SE1 7EH UK; 2 Department of Radiology, Centre Hospitalier Universitaire de Liege, Cour des Mineurs 5D, 4000 Liege, Belgium; 3 Department of Clinical Oncology, Guys and St Thomas’ Hospital NHS Trust, London, UK; 4 King’s College London & Guy’s and St Thomas’ PET Centre, St Thomas’ Hospital, London, SE1 7EH UK

**Keywords:** Breast cancer, Bone metastases, Heterogeneity, [^18^F]fluoride PET/CT, [^18^F]FDG PET/CT

## Abstract

**Purpose:**

To establish whether first-order statistical features from [^18^F]fluoride and 2-deoxy-2-[^18^F] fluoro-d-glucose ([^18^F]FDG) positron emission tomography/x-ray computed tomography (PET/CT) demonstrate incremental value in skeletal metastasis response assessment compared with maximum standardised uptake value (SUV_max_).

**Procedures:**

Sixteen patients starting endocrine treatment for *de novo* or progressive breast cancer bone metastases were prospectively recruited to undergo [^18^F]fluoride and [^18^F]FDG PET/CT scans before and 8 weeks after treatment. Percentage changes in SUV parameters, metabolic tumour volume (MTV), total lesion metabolism (TLM), standard deviation (SD), entropy, uniformity and absolute changes in kurtosis and skewness, from the same ≤ 5 index lesions, were measured. Clinical response to 24 weeks, assessed by two experienced oncologists blinded to PET/CT imaging findings, was used as a reference standard and associations were made between parameters and progression free and overall survival.

**Results:**

[^18^F]fluoride PET/CT: In four patients (20 lesions) with progressive disease (PD), TLM and kurtosis predicted PD better than SUV_max_ on a patient basis (4, 4 and 3 out of 4, respectively) and TLM, entropy, uniformity and skewness on a lesion basis (18, 16, 16, 18 and 15 out of 20, respectively). Kurtosis was independently associated with PFS (*p* = 0.033) and OS (*p* = 0.008) on Kaplan-Meier analysis. [^18^F]FDG PET: No parameter provided incremental value over SUV_max_ in predicting PD or non-PD. TLM was significantly associated with OS (*p* = 0.041) and skewness with PFS (*p* = 0.005). Interlesional heterogeneity of response was seen in 11/16 and 8/16 patients on [^18^F]fluoride and [^18^F]FDG PET/CT, respectively.

**Conclusion:**

With [^18^F]fluoride PET/CT, some first-order features, including those that take into account lesion volume but also some heterogeneity parameters, provide incremental value over SUV_max_ in predicting clinical response and survival in breast cancer patients with bone metastases treated with endocrine therapy. With [^18^F]FDG PET/CT, no first-order parameters were more accurate than SUV_max_ although TLM and skewness were associated with OS and PFS, respectively. Intra-patient heterogeneity of response occurs commonly between metastases with both tracers and most parameters.

## Introduction

Skeletal metastases are common in patients with advanced breast cancer and are associated with significant morbidity [1]. With the introduction of new systemic therapies that improve survival time, early detection and response assessment of skeletal metastases has become more important. Varied tumour response to treatment is undoubtedly an important factor in the clinical outcome, accentuating the need to have reliable measures to monitor patients for early disease progression in order to allow timely discontinuation of ineffective treatment.

Conventionally, bone scintigraphy has been used to assess breast cancer bone metastases but has certain limitations when assessing treatment response [2]. Molecular and functional imaging can improve diagnosis and treatment response assessment of breast cancer bone metastases [3]. Standardised uptake value (SUV) on positron emission tomography/computed tomography (PET/CT) has been used as a standard semi-quantitative method for monitoring treatment response but other non-heterogeneity parameters such as metabolic tumour volume (MTV) and tumour lesion metabolism (TLM) have also been used to measure metabolic activity within the tumour [4, 5]. Several retrospective studies using 2-deoxy-2-[^18^F] fluoro-d-glucose ([^18^F]FDG) PET/CT, mainly focusing on osseous response to treatment, have established that a change in SUV_max_ can predict disease response or progression [6–8] and a feasibility study has also shown that [^18^F]fluoride PET may be useful in evaluating treatment response in breast cancer [9]. Despite this, there is limited evidence to support the use of either tracer in routine clinical practice. Measuring volumetric parameters or heterogeneity of tracer activity has also been shown to have incremental predictive or prognostic value in a number of cancers [10–21]. First-order statistics measure global properties of a tumour from individual voxel values and can be obtained from the histogram of voxel intensities and are most commonly used [22], but there are no reports on the use of first-order heterogeneity parameters in evaluating treatment response assessment of breast cancer bone metastases using [^18^F]fluoride or [^18^F]FDG PET.

The hypothesis of this study was that global first-order features derived from skeletal metastases, some of which describe heterogeneity in bone metastases, may be a better predictor of response to treatment in comparison with SUV_max_.

The objective of this study was to extract first-order features on both [^18^F]FDG (tumour-specific radiotracer that targets glucose metabolism) and [^18^F]fluoride (bone-specific tracer that targets osteoblast activity and local blood flow) PET/CT images in breast cancer bone metastases, at baseline and 8 weeks after endocrine treatment, and to compare their ability to predict treatment response determined by a clinical reference standard as well as survival with the most commonly described parameter, SUV_max_.

## Materials and Methods

### Participants

Sixteen female breast cancer patients (mean age 51.6, range 40–79 years) starting endocrine treatment for *de novo* (*n* = 5) or progressive bone metastases (*n* = 11) from an ongoing prospective single-centre exploratory study were included. The endocrine treatments used were letrozole (*n* = 12), tamoxifen (*n* = 2), everolimus/exemestane (*n* = 1) and anastrazole (*n* = 1). Apart from two patients who had small volume lung and liver metastases, all other patients had bone-only disease. [^18^F]fluoride and [^18^F]FDG PET/CT scans were acquired before and 8 weeks after starting treatment. The study was approved by a Research Ethics Committee and the Administration of Radioactive Substances Advisory Committee and all patients signed an informed consent form at the time of recruitment.

### [^18^F]FDG PET/CT and [^18^F]fluoride PET/CT Image Acquisition

After injection of [^18^F]FDG (mean 348 ± 18 MBq), PET/CT scans commenced after an uptake time of 60 min. On a separate day following injection of [^18^F]fluoride (mean 228 ± 15 MBq), scans were performed after an uptake time of 60 min. Imaging comprised a static PET/CT scan using a GE Discovery 710 PET/CT scanner (GE Healthcare, Chicago, USA). Each scan covered the base of the skull to mid-thigh, with an axial field-of-view of 15.7 cm and an 11-slice overlap between bed positions. A low-dose CT scan (140 kV, 10 mA, 0.5 s rotation time and 40 mm collimation) was performed at the start of imaging to provide attenuation correction and an anatomical reference. PET scan duration was set to 3 min per bed position.

PET image reconstruction included standard scanner-based corrections for radiotracer decay, scatter, randoms and dead-time. Emission sinograms were reconstructed with a time-of-flight ordered subset expectation maximisation (OSEM) algorithm (2 iterations, 24 subsets), with a 256 × 256 matrix and a 4-mm full-width at half-maximum (FWHM) Gaussian post-reconstruction smoothing filter on the scanner front end, available from the manufacturer.

### Parameter Analysis

**Fig. 1.**
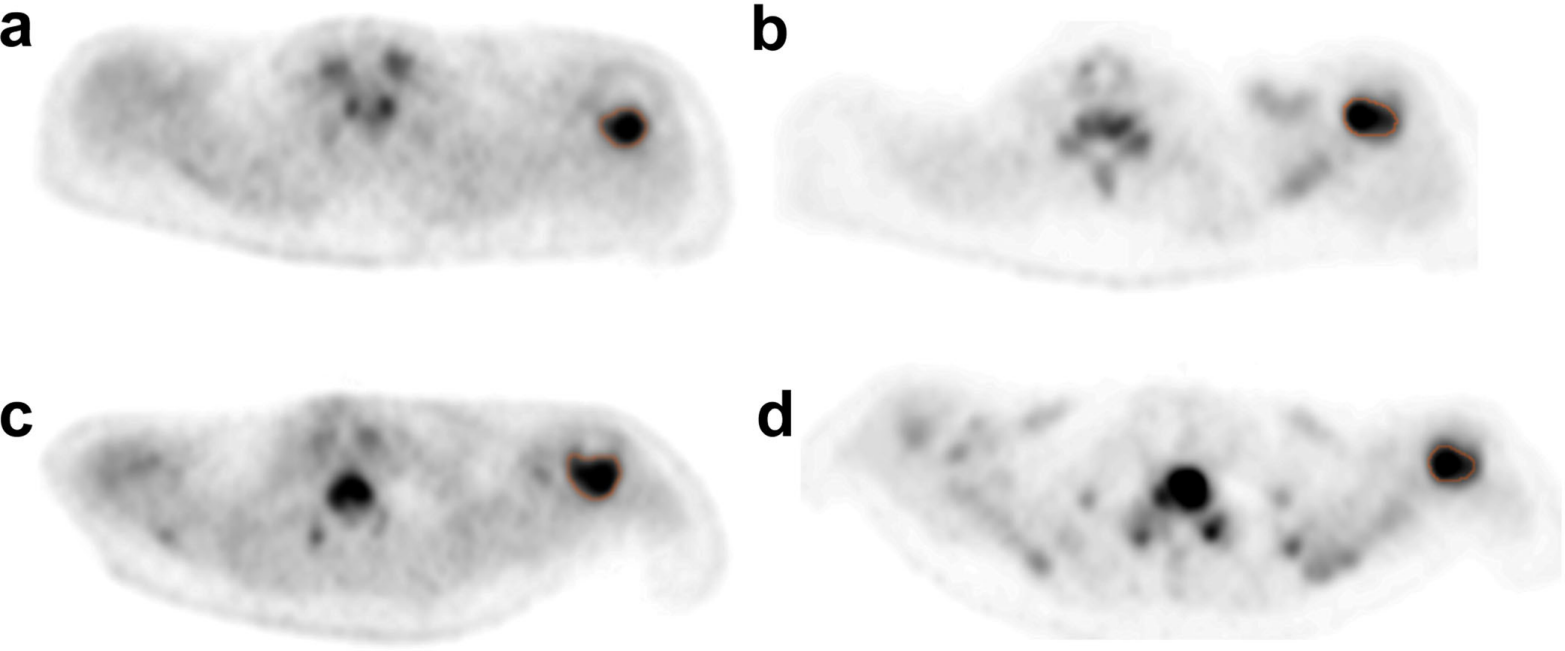
Figure demonstrating regions of interest in the left humerus in a patient with clinical progressive disease. **a**, **c** [^18^F]FDG and **b**, **d** [^18^F]fluoride PET transaxial slices **a**, **b** before and **c**, **d** 8 weeks after treatment. [^18^F]FDG SUV_max_ 14.6 at baseline and 15.4 at 8 weeks. [^18^F]fluoride SUV_max_ 37.7 at baseline, 56.5 at 8 weeks and 82.4 at 12 weeks (not shown).

**Fig. 2.**
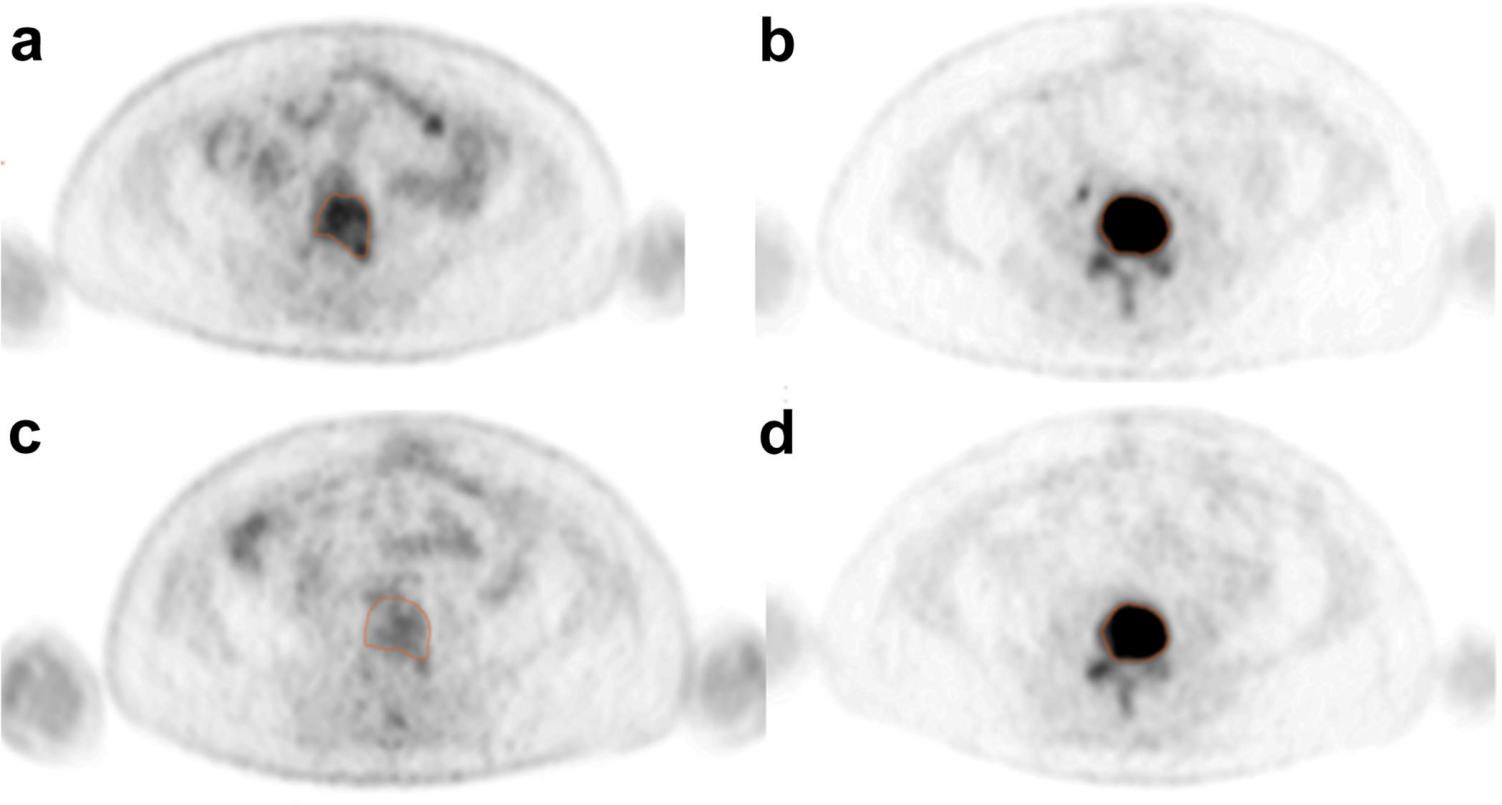
Figure demonstrating regions of interest in L4 in a patient with non-progressive disease (partial response). **a**, **c** [^18^F]FDG and **b**, **d** [^18^F]fluoride PET transaxial slices **a**, **b** before and **c**, **d** 8 weeks after treatment. [^18^F]FDG SU_Vmax_ 8.4 at baseline and 4.4 at 8 weeks. [^18^F]fluoride SUV_max_ 72.1 at baseline and 46.5 at 8 weeks.

Up to five of the most active (SUV ≥ 10) [23] and largest (≥ 1 cm diameter) lesions were first identified for analysis on [^18^F]fluoride scans in each subject. Regions of interest (ROIs) were delineated around the same metastasis on the static [^18^F]fluoride and [^18^F]FDG PET/CT scans by an oncologist and radiologist working in consensus (Figs. 1 and 2). Image heterogeneity analysis was performed using in-house quantitative analysis software implemented in MATLAB (Mathworks, Natick, MA, USA). First-order statistics derived from regional geometry and the histogram distribution of voxel intensities (standard deviation (SD), entropy, uniformity, kurtosis and skewness) on both [^18^F]FDG and [^18^F]fluoride PET scans were calculated as well as non-heterogeneity parameters such as SUV_max_, SUV_mean_, SUV_peak_, TLM and MTV. All parameters on both PET scans were calculated for the same lesions at baseline and 8 weeks, and changes in the values of these parameters from baseline were used for statistical analyses. The tumour volumes were generally small ((mean volume = 7.1 cm^3^ (SD = 8.3) on [^18^F]fluoride PET/CT and (mean volume = 5.7 cm^3^ (SD = 5.9) on [^18^F]FDG PET/CT)); therefore, in order to avoid bias from small volumes, second and high-order texture features were not calculated [24, 25]. We also analysed changes in SUV_max_ between lesions in each individual patient to assess the degree of interlesional heterogeneity of response with both tracers. Interlesional heterogeneity was defined when a metastasis showed a change in parameter that was opposite to the clinical reference standard.

Two experienced oncologists working in consensus, blinded to PET/CT findings, determined clinical response based on standard imaging including bone scans and contrast-enhanced CT, clinical assessment, including pain scores (using brief pain inventory questionnaire), as well as alkaline phosphatase and carcinoma antigen 15-3 serology up to 24 weeks after the start of treatment or until progression, whichever came first and was used as a reference standard (Table 1). The changes in clinical parameters were used in patient assessment and none of the parameters were used in isolation. Any discrepancy was reviewed by a third clinician and only one went to a third reader. Assessment decisions were made on bone-only disease, given no soft tissue disease in the majority of this group, so soft tissue response (Response Evaluation Criteria In Solid Tumours (RECIST)) was not relevant in our studied population. Patients were grouped into progressive disease (PD) and non-progressive disease (non-PD = partial response (PR) and stable disease (SD)). PR and SD patients were assessed together as clinical management is rarely different in these two groups.

**Table 1:** Different parameters used in each patient for categorisation into PD or non-PD

Patient	Disease status	ALP (alkaline phosphatase)	Ca-153	Pain score	Clinical assessment	Bone scan	CT scan
1	PD	Increased	Stable	Worse	New bone pain	Not done	Increase in number of lesions by 24 weeks
2	PD	Increased	Increased	Stable	Weight loss, vomiting	Increase in number of lesions by 24 weeks	Increase in number of lesions by 24 weeks
3	PD	Stable	Increased	Worse	New bone pain	Increase in number of lesions by 24 weeks	Increase in number of lesions by 24 weeks
4	PD	Increased	Stable	Worse	Worsening bone pain	Increase in number of lesions by 24 weeks	Not done
5	Non-PD	Increased	Increased	Better	Better	No change	No change
6	Non-PD	Stable	Decreased	Better	Stable symptoms	Not done	No change
7	Non-PD	Stable	Stable	Better	Asymptomatic	No change	No change
8	Non-PD	Stable	Stable	Better	Better	No change	Disappearance of some lesions
9	Non-PD	Better	Better	Better	Stable symptoms	Not done	Disappearance of dome lesions
10	Non-PD	Better	Stable	Better	Stable symptoms	Not done	No change
11	Non-PD	Stable	Stable	Better	Asymptomatic	No change	No change
12	Non-PD	Stable	Stable	Better	Stable symptoms	No change	
13	Non-PD	Stable	Better	Stable	Asymptomatic	No change	Disappearance of some lesions
14	Non-PD	Better	Better	Better	Stable symptoms	No change	Disappearance of some lesions
15	Non-PD	Stable	Better	Better	Stable symptoms	Disappearance of some lesions	Disappearance of some lesions
16	Non-PD	Better	Stable	Better	Stable symptoms	No change	No change

### Statistical Analysis

Statistical analysis was performed using SPSS for windows version 24 (IBM SPSS Statistics 24). After testing for normality, parametric or nonparametric tests were applied to each set of data. Data that were normally distributed were expressed as a mean and standard deviation and evaluated using the paired *t* test. Data that were not normally distributed were expressed as median and range and evaluated using Wilcoxon signed rank test or Mann-Whitney *U* test. For all statistical tests, a *P* value of ≤ 0.05 was considered statistically significant.

On both [^18^F]fluoride and [^18^F]FDG PET/CT scans, optimum threshold values were established with receiver operating characteristic analysis, maximising the sum of sensitivity and specificity by measuring associated areas under the ROC curves as there are no established criteria for these tracers with endocrine treatment, although ± 25 % has been suggested for [^18^F]FDG post-chemotherapy [26] (Tables 2 and 3). On this basis, we also used a 25 % cut-off for the SUV parameters on [^18^F]FDG (adapted from the EORTC criteria) and [^18^F]fluoride PET/CT scans, acknowledging that these criteria were described for [^18^F]FDG PET/CT. The values of percentage (%) changes in SUV_max_, SUV_mean_, SUV_peak_, TLG, MTV, entropy, uniformity and absolute changes in skewness and kurtosis, on [^18^F]fluoride and [^18^F]FDG PET/CT scans after 8 weeks of treatment, were calculated for all patients.

**Table 2:** Changes in parameter values for PD and non-PD after 8 weeks of treatment on [^18^F]fluoride PET/CT with associated optimum and 25 % cut-off values and AUC

	PD (20 lesions/4 patients)							Non-PD (52 lesions, 12 patients)					*P* value for PD *vs* non-PD
	Median change (%)	Optimal cut-off (ROC curve analysis)		Number of lesions identified (↑ or ↓)		Number of patients correctly identified (↑ or ↓)		Median change (%)	Number of lesions identified (↑ or ↓)		Number of patients identified (↑ or ↓)		
		Value	AUC	Opt cut-off	25 % cut-off	Opt cut-off	25 % cut-off		Opt cut-off	25 % cut-off	Opt cut-off	25 % cut-off	
SUVmax (%)	− 3.0	− 15.1	0.67	15 ↑	7 ↑	3 ↑	2 ↑	− 20.1	29 ↓	47 ↓	5 ↓	9 ↓	0.021
SUV_mean_ (%)	15.1	− 2.6	0.75	14 ↑	6 ↑	3 ↑	2 ↑	− 16.0	38 ↓	46 ↓	8 ↓	9 ↓	0.001
SUV_peak_ (%)	3.0	− 3.9	0.72	13↑	8 ↑	3 ↑	2 ↑	− 17.6	31 ↓	46 ↓	7 ↓	9 ↓	0.003
MTV (%)	− 2.5	19.0	0.51	14 ↓		0		1.1	44 ↓		10 ↓		0.806
TLM (%)	9.2	− 19.0	0.67	18 ↑		4 ↑		− 11.2	26 ↑		8 ↑		0.023
SD (%)	4.9	9.2	0.67	10 ↑		2 ↑		− 25.4	43↓		8 ↓		0.022
Ent (%)	1.3	0.6	0.67	16 ↑		3 ↑		− 0.5	37 ↓		5 ↓		0.020
Unif (%)	− 6.6	1.8	0.69	16 ↓		3 ↓		2.9	31 ↑		7 ↑		0.012
Kurt (abs)	− 0.1	− 0.005	0.66	14 ↓		4 ↓		0.04	31 ↑		8 ↑		0.034
Skew (abs)	− 0.05	0.1	0.5	18 ↓		3 ↓		0.01	37 ↓		11 ↓		0.277

*MTV* metabolic tumour volume, *TLM* tumour lesion metabolism, *SD* standard deviation, *Ent* entropy, *Unif* uniformity, *Kurt* kurtosis, *Skew* skewness, *abs* absolute change, *%* percentage change, ↑ ≥ the cut-off value, ↓ < the cut-off value

**Table 3:** Changes in parameter values for PD and non-PD after 8 weeks of treatment on [^18^F]FDG PET/CT with associated optimum and 25 % cut-off values and AUC

	PD (20 lesions/4 patients)							Non-PD (52 lesions/12 patients)					*P* value for PD *vs* non-PD
	Median change	Optimal cut-off (ROC curve analysis)		Number of lesions identified (↑ or ↓)		Number of patients identified (↑ or ↓)		Median change	Number of lesions identified (↑ or ↓)		Number of patients identified (↑ or ↓)		
		Value	AUC	Opt cut-off	25 % cut-off	Opt cut-off	25 % cut-off		Opt cut-off	25 % cut-off	Opt cut-off	25 % cut-off	
SUV_max_ (%)	− 2.2	− 20.6	0.76	18 ↑	2 ↑	4 ↑	0 ↑	− 27.2	33 ↓	52 ↓	7 ↓	12 ↓	0.001
SUV_mean_ (%)	0.4	− 21.7	0.77	18 ↑	1 ↑	4 ↑	0 ↑	− 24.2	28 ↓	52 ↓	4 ↓	12 ↓	0.001
SUV_peak_ (%)	0.6	− 15.0	0.76	17 ↑	2 ↑	3 ↑	0 ↑	− 24.7	33 ↓	52 ↓	6 ↓	12 ↓	0.001
MTV (%)	10.7	− 14.1	0.70	17 ↑		4 ↑		− 19.7	30 ↓		6 ↓		0.006
TLM (%)	4.2	− 24.1	0.76	18 ↑		4 ↑		− 40.2	33 ↓		6 ↓		0.001
SD (%)	4.1	− 23.4	0.72	16 ↑		4 ↑		− 33.5	33 ↓		7 ↓		0.004
Ent (%)	− 0.7	− 2.2	0.55	15 ↑		4 ↑		− 1.1	33 ↑		7 ↑		0.458
Unif (%)	2.8	5.8	0.59	15 ↓		3 ↓		4.4	27 ↓		6 ↓		0.235
Kurt (abs)	0.1	0.2	0.51	16 ↓		4 ↓		0.09	29 ↓		7 ↓		0.826
Skew (abs)	0.05	− 0.13	0.55	14 ↑	0	4 ↑	0	− 0.03	29 ↑		6 ↑		0.517

*MTV* metabolic tumour volume, *TLM* tumour lesion metabolism, *SD* standard deviation, *Ent* entropy, *Unif* uniformity, *Kurt* kurtosis, *Skew* skewness, *abs* absolute change, *%* percentage change, ↑ ≥ the cut-off value, ↓ < the cut-off value

Kaplan-Meier analysis was performed using the median value for each parameter to dichotomise the results with differences in the curves tested with the log-rank test. Progression free survival (PFS) was defined as the time between the date of the start of endocrine treatment and the date of disease progression and overall survival (OS) was calculated from the start of endocrine treatment to the date of death or until censoring on the date of the last follow-up.

## Results

There was a total of 16 patients (72 lesions). By the clinical reference standard, 4 patients (20 lesions) had PD at or before 24 weeks and 12 patients (52 lesions) non-PD at 24 weeks. Patients were followed up from between 12 and 49 (median 31.5) months. Four patients died during the follow-up period and all 16 patients progressed at between 2 and 32 (median 11.3) months.

Using the cut-offs that maximised sensitivity and specificity from ROC analysis (Tables 2 and 3), on [^18^F]fluoride PET/CT, in all 16 patients, MTV, TLM, kurtosis and skewness performed better than SUV_max_ on a patient basis (10, 12, 12, 11 and 8 out of 16, respectively) and SD, entropy, uniformity, kurtosis and skewness on a lesion basis (53, 53, 47, 45, 55 and 44 out of 72, respectively). In the four patients with PD, TLM and kurtosis predicted PD better than SUV_max_ on a patient basis (4, 4 and 3 out of 4, respectively) and TLM, entropy, uniformity and skewness on a lesion basis (18, 16, 16, 18 and 15 out of 20, respectively) (Table 2). In the 12 patients with non-PD, [^18^F]fluoride MTV, TLM, SD, uniformity, kurtosis and skewness predicted non-PD better than SUV_max_ on a patient basis (10, 8, 8, 7, 8, 11 and 5 out of 12, respectively) and MTV, SD, entropy, uniformity and skewness on a lesion basis (44, 43, 37, 31, 37 and 30 out of 52, respectively (Table 2). On [^18^F]FDG PET/CT, no parameter provided incremental value over SUV_max_ overall or in predicting PD or non-PD (Table 3).

In PD and non-PD, on both [^18^F]fluoride PET/CT and [^18^F]FDG PET/CT scans, median % change in first-order features was not statistically significantly different from the median % change in the SUV parameters. On a per lesion basis, on [^18^F]fluoride PET/CT, median % changes in SUV_max_, SUV_mean_, SUV_peak_, TLM, SD and entropy were significantly higher in PD than non-PD (− 3 *vs* − 20.1 % (*p* = 0.021), 15.1 *vs* − 16 % (*p* = 0.001), 3 *vs* − 17.6 % (*p* = 0.003), 9.2 *vs* − 11.2 % (*p* = 0.023), 4.9 *vs* − 25.4 % (*p* = 0.022), 1.3 *vs* − 0.5 % (*p* = 0.020), respectively) and median % changes in uniformity and kurtosis were significantly lower in PD than non-PD (− 6.6 *vs* 2.9 % (*p* = 0.012), − 0.1 *vs* 0.04 % (*p* = 0.034), respectively) (Table 2). On [^18^F]FDG PET/CT, the median % changes in SUV_max_, SUV_mean,_ SUV_peak,_ MTV, TLM and SD were significantly higher in patients with PD than non-PD (− 2.2 *vs* − 27.2 % (*p* = 0.001), 0.4 *vs* − 24.2 % (*p* = 0.001), 0.6 *vs* − 24.7 % (*p* = 0.001), 10.7 *vs* − 19.7 % (*p* = 0.006), 4.2 *vs* − 40.2 % (*p* = 0.001), 4.1 *vs* − 33.5 % (*p* = 0.004), respectively) (Table 3). Other changes were not statistically significant.

Interlesional heterogeneity of response was seen in 11/16 and 8/16 patients on [^18^F]fluoride and [^18^F]FDG PET/CT, respectively. For [^18^F]fluoride, there was a statistically significant difference between the 15 lesions that showed an increase in SUV_max_, in patients with PD and the 22 lesions that showed an increase in SUV_max_ in patients with non-PD for entropy (*p* = 0.028), uniformity (*p* = 0.008) and kurtosis (0.033). No non-PD patient showed new lesions at 8 weeks with either [^18^F]FDG or [^18^F]fluoride PET/CT.

**Fig. 3.**
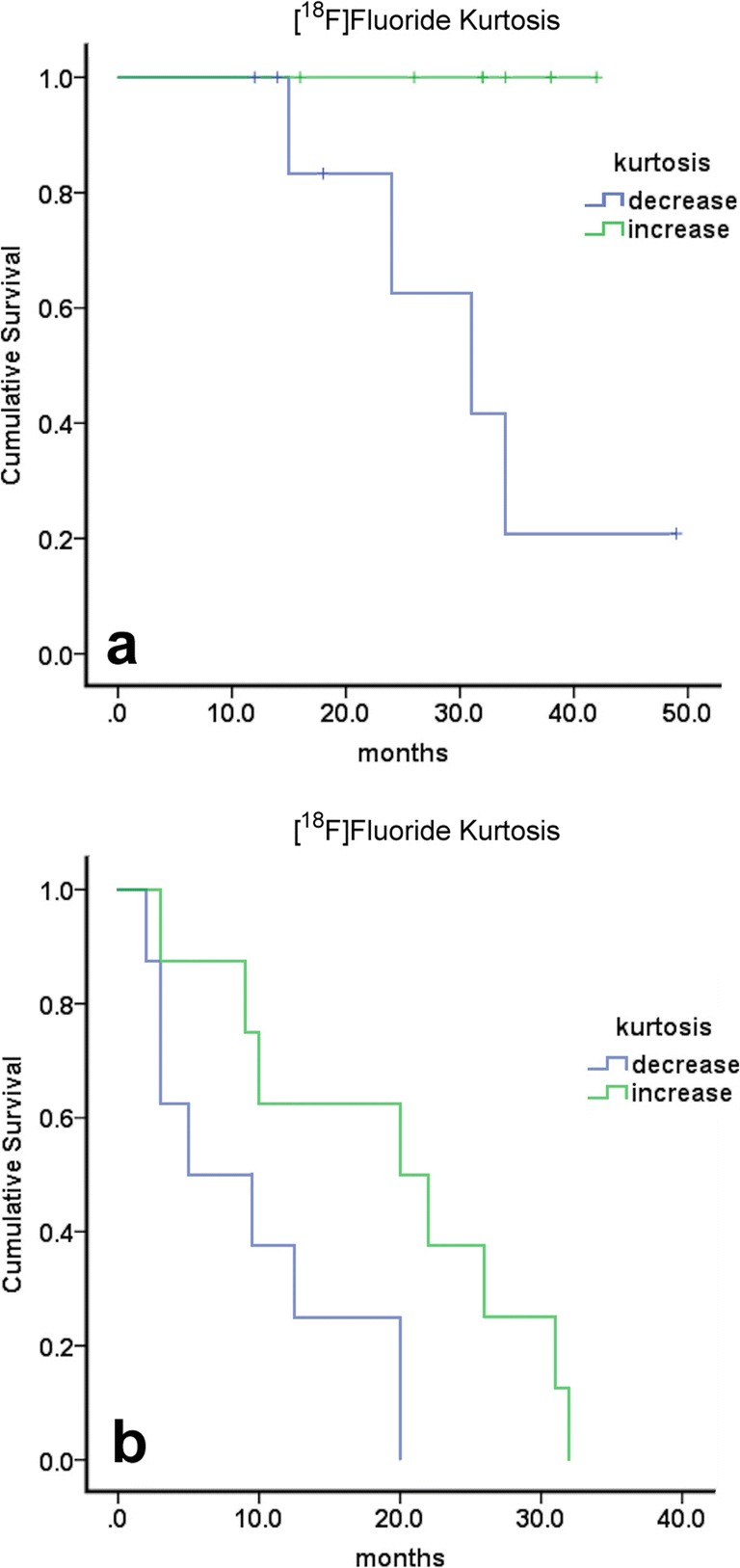
Kaplan-Meier graphs showing worse **a** OS and **b** PFS in patients with negative changes in kurtosis less than the median (*p* = 0.008 and 0.033, respectively) for [^18^F]fluoride PET/CT scans.

Kaplan-Meier analysis showed that on [^18^F]fluoride PET/CT, at 8 weeks, change in kurtosis had a statistically significant association with PFS (*p* = 0.033) and OS (*p* = 0.008) (Fig. 3a, b). On [^18^F]FDG PET/CT, change in TLM was significantly associated with OS (*p* = 0.041) and skewness with PFS (*p* = 0.005).

## Discussion

Breast cancer is commonly associated with skeletal metastases and early evaluation of response or progression to treatment is vital to the optimisation of patients’ clinical management. To our knowledge, this is the first report that has evaluated several first-order statistical features, including some heterogeneity parameters, for early treatment response assessment of breast cancer bone metastases compared to standard SUV measures using [^18^F]fluoride and [^18^F]FDG PET/CT.

**Fig. 4.**
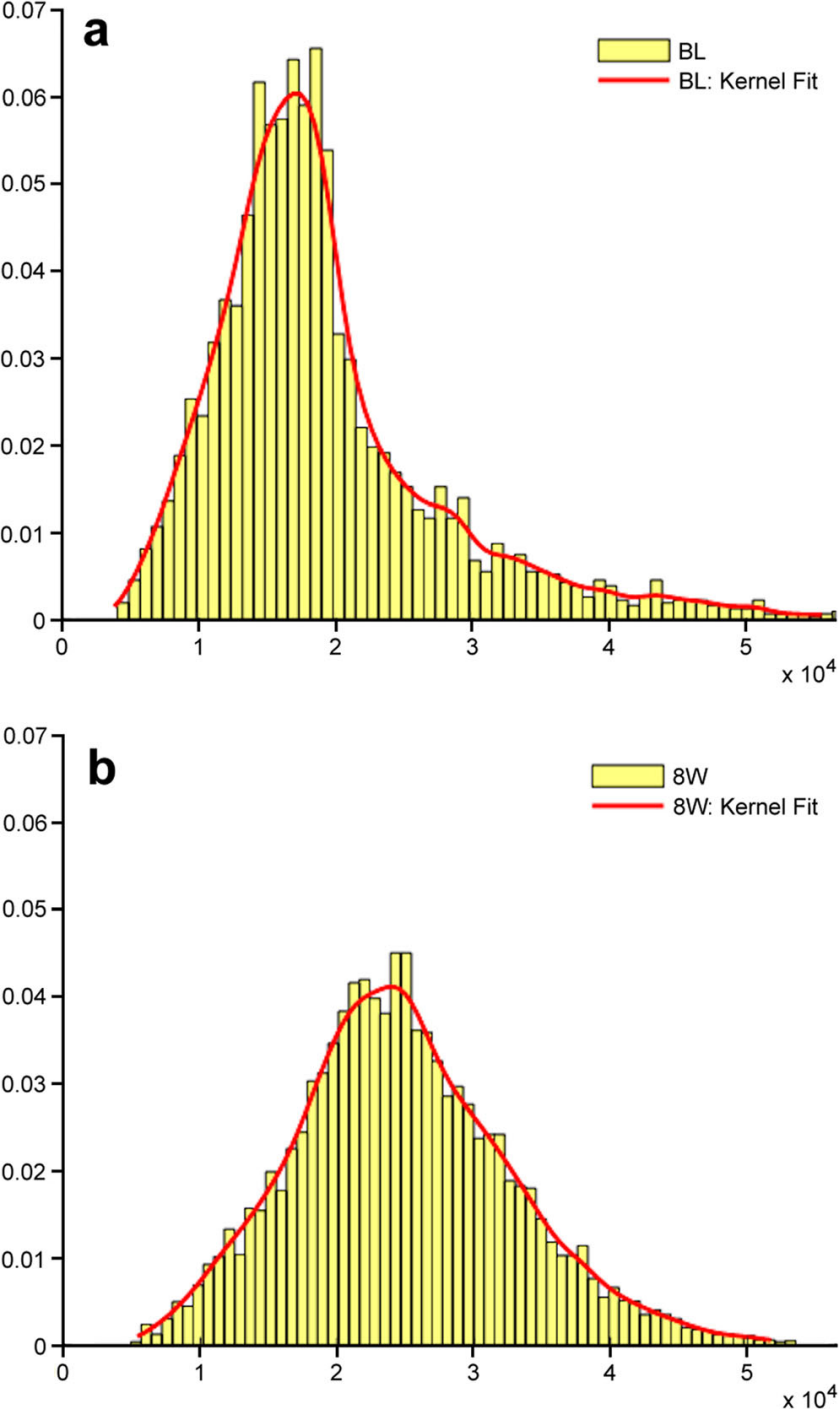
Voxel intensity histograms at **a** baseline (BL) and **b** 8 weeks (8W) with kernel fits of a metastasis in a patient with PD on [^18^F]fluoride PET/CT. As well as an increase in intensity of voxels (SUV_max_ rose from 32.7 to 38.0 and SUV_mean_ from 12.8–15.3), the histogram becomes less peaked (kurtosis decreased from 5.6 to 3.1).

For [^18^F]fluoride PET/CT, several first-order global parameters showed superiority over SUV_max_, either on a patient-based or lesion-based analysis in predicting PD, including volume-based parameters (MTV, TLM) and heterogeneity parameters (entropy, uniformity, kurtosis and skewness). Additionally, kurtosis was associated with both PFS and OS. Whilst recognition of PD is of most clinical importance, as these patients will need an early transition to second-line therapy, the majority of first-order parameters were also better than SUV_max_ at predicting non-PD. The observed changes in patients with PD were as expected, *i.e.,* an increase in activity, volume and/or heterogeneity. In particular, a decrease in kurtosis was also associated with PFS and OS. This relates to an increase in spread (less peakedness) of the voxel intensity histogram (Fig. 4a, b) or greater “heterogeneity” in voxel values within lesions.

Whilst changes in SUV and volume parameters, as well as kinetic analysis, have been reported for monitoring therapy with [^18^F]fluoride PET in bone metastases [9, 27–29], to our knowledge, there are no data describing superiority of heterogeneity parameters in this situation. However, first-order heterogeneity parameters have shown predictive and prognostic ability in other cancers with [^18^F]FDG PET/CT and increased heterogeneity is usually associated with more aggressive tumours and poor treatment response [11, 13, 21, 30].

For [^18^F]FDG PET/CT, no parameter performed better than SUV_max_ in predicting response, although an increase in TLM and skewness (shift of histogram to the right with more high intensity voxels) was associated with poor OS and PFS, respectively, whereas SUV_max_ was not prognostic. In accordance with our findings, changes in SUV_max_ have previously been shown to be valuable in assessing treatment response in breast cancer skeletal metastases [7, 8].

Heterogeneity of response between metastases is a recognised phenomenon [31] and occurred in 11/16 of our patients with [^18^F]fluoride and 8/16 with [^18^F]FDG. The more frequent occurrence with [^18^F]fluoride may be partly explained by the flare phenomenon whereby a temporary increase in osteoblastic activity can occur in healing metastases in non-PD patients [32–34]. Despite this and the fact that uptake of the two tracers is dependent on differing underlying biological processes (tumour cell glucose metabolism with [^18^F]FDG and osteoblastic mineralisation of bone with [^18^F]fluoride), both tracers offered predictive and prognostic information at a level that would be of clinical utility on a per patient basis. We also observed that entropy, uniformity and kurtosis were significantly different in the 15/20 lesions with a concordant increase in SUV_max_ in PD patients compared to a discordant increase in the 22/52 lesions in patients with non-PD, the latter that could be attributed to the flare phenomenon. This would require further prospective validation but the potential to differentiate an increase in uptake due to true progression from the flare phenomenon at 8 weeks would be of great clinical utility when using [^18^F]fluoride PET to measure early response, overcoming one of the limitations of bone-specific imaging.

Limitations of this study include a relatively small number of patients, although we were able to include a large number of individual metastases in the analysis (*n* = 72). In addition, there was a smaller number of patients with PD (*n* = 4) compared to non-PD which may have introduced an element of statistical bias. Whilst all patients had similar treatment, *i.e.,* endocrine therapy, treatment regimens were not exactly the same and there was probably heterogeneity in response. Nevertheless, the main objective of this exploratory study was to measure response rather than treatment-specific effects. Though our study was prospective, these findings deserve further evaluation in larger cohorts as well as in the assessment of different types of therapy and bone metastases from other cancers. Whilst there is no gold standard for determining treatment response in bone metastases, our clinical reference standard was made as robust and clinically relevant as possible by including clinical findings, conventional imaging, biochemistry and tumour markers up to 24 weeks assessed by two oncologists in consensus and we were also able to include a survival analysis as an objective assessment of the measured parameters. Whilst criteria for response assessment have previously been reported for [^18^F]FDG SUV_max_ or SUV_peak_ [3, 35], these criteria have not been applied to other first-order parameters and so we used optimal cut-offs in this exploratory study in addition to 25 % cut-offs for [^18^F]FDG and [^18^F]fluoride conventional SUV parameters. We acknowledge that repeatability of first-order texture features is variable and that the optimal cut-offs for some parameters that have been reported as showing lower repeatability, such as uniformity and skewness [36], may be within the limits of repeatability measurements. Several novel parameters still performed better, even when an optimal SUV_max_ cut-off was used for comparison.

## Conclusions

Our exploratory data demonstrate that certain first-order statistical features from [^18^F]fluoride and [^18^F]FDG PET, related to volume and heterogeneity, may provide incremental value over SUV_max_ in the prediction of treatment response and survival in breast cancer bone metastases treated with endocrine therapy, a finding that deserves confirmation in further prospective evaluation in future studies. In addition, with [^18^F]fluoride some heterogeneity parameters can potentially differentiate an increase in SUV_max_ due to flare from that due to progression of disease. These findings may be of potential clinical utility as the prediction of early PD helps oncologists decide whether an earlier switch to more effective therapies is required, whereas non-PD patients would generally continue the same treatment if there were no significant toxicities. We also observed that intra-patient heterogeneity of response occurs commonly between metastases with both tracers and most parameters.
